# Suppression of cucumber stachyose synthase gene (*CsSTS*) inhibits phloem loading and reduces low temperature stress tolerance

**DOI:** 10.1007/s11103-017-0621-9

**Published:** 2017-06-12

**Authors:** Jianguo Lü, Xiaolei Sui, Si Ma, Xin Li, Huan Liu, Zhenxian Zhang

**Affiliations:** 1 Beijing Key Laboratory of Growth and Developmental Regulation for Protected Vegetable Crops, College of Horticulture, China Agricultural University, Beijing, 100193 China; 2 College of Agricultural and Biological Sciences, Dali University, Dali, 671003 Yunnan China

**Keywords:** Cucumber, Low temperature stress, Phloem, Stachyose, Stachyose synthase

## Abstract

Stachyose is the main transporting sugar in phloem of Raffinose family oligosaccharides-transporting species. Stachyose synthase (STS) is a key enzyme for stachyose biosynthesis, but the gene encoding STS is poorly characterized in cucumber (*Cucumis sativus* L.), which is a model plant for studying stachyose metabolism and phloem function. In this research, stachyose synthase gene (*CsSTS*) from cucumber was isolated and its physiological functions were analyzed. *CsSTS* expressed mainly in the phloem of the minor veins in mature leaves and localized to companion cells. Reverse genetics with *CsSTS* RNAi lines revealed obviously reductions in STS activity and stachyose content along with a small amount of starch accumulation in leaves, suggesting that *CsSTS* is involved in phloem loading of cucumber leaves. After 6 °C low temperature stress, malondialdehyde content and electrical conductivity increased, especially in *CsSTS*-RNAi plants. But *CsSTS* expression was up-regulated, STS activity and stachyose level increased, the activities of reactive-oxygen-scavenging enzyme in cucumber seedlings improved significantly and starch accumulation reduced, especially in *CsSTS*-OE lines. These results demonstrate clearly that *CsSTS* is involved in phloem loading, carbohydrate distribution and tolerance of cucumber seedlings to low temperature stress.

**Electronic supplementary material:**

The online version of this article (doi:10.1007/s11103-017-0621-9) contains supplementary material, which is available to authorized users.

## Introduction

The ability of plants to translocate a portion of photosynthetically derived carbon for storage allows them to grow and survive in a variety of environments and under many different conditions (Unda et al. 2012). Phloem loading is a process by which sugars and other products of photosynthesis are exported from mesophyll cells into the sieve elements (SE) and companion cells (CC) within minor veins of plants (Gamalei 2007; Turgeon and Wolf 2009). In most plants, sucrose serves as the primary transported sugar. In addition to sucrose, the Raffinose family oligosaccharides (RFOs) including stachyose and raffinose, are used in some plant species such as *Cucurbitaceae* and *Scrophulariaceae* for long-distance transport (Hendrix 1982; Mitchell and Mador 1^992^). In some other species, such as *Rosaceae*, sugar alcohol (sorbitol or mannitol) is the main component for phloem transport next to sucrose (Loescher and Everard 2000; Noiraud et al. 2001).

The three principal mechanisms of phloem loading include one apoplastic and two symplastic pathways (Rennie and Turgeon 2009; Slewinski and Braun 2010). The two symplastic loading pathways identified for sugar are the thermodynamically active and passive pathways (Reidel et al. 2009; Rennie and Turgeon 2009; Turgeon 2010). Hence, it is generally accepted that three strategies or types of phloem loading can be defined in the minor veins of plant: passive symplastic diffusion, active symplastic polymer trapping, and active apoplastic loading.

Passive symplastic loading (diffusion) mechanism in some species, such as a large number of woody species, especially trees, is driven by diffusion along a concentration gradient to enter phloem sieve tubes through the plasmodesmata from the high sucrose concentrations (in some cases sugar alcohols) in mesophyll cells, and requires no energy (Rennie and Turgeon 2009; Slewinski and Braun 2010). The active process of symplastic polymer trapping mechanism involves diffusion of assimilates synthesized in the mesophyll cells from the bundle sheath cells through abundant plasmodesmata into specialized companion cells (intermediary cells, i.e., IC), where the diffused assimilates are converted into the larger oligosaccharides (RFOs), raffinose and stachyose. A remarkable feature of this mechanism is the size exclusion limits of those plasmodesmata between the IC and the mesophyll cells allow diffusion of sucrose into the IC, but do not permit diffusion of the larger molecular weight sugars, RFOs, in the opposite direction. Polymer trapping is a thermodynamically active mechanism since energy is used to create a concentration difference between the mesophyll cells and the SE-CC complex (Turgeon 2010).

In active apoplastic loading pathway, sucrose synthesized in the mesophyll cells moves into phloem parenchyma cells by symplastic transfering and is then loaded into the phloem by specific transport proteins (Ayre 2011). Because sucrose is exported against the concentration gradient, this importation process requires energy (Giaquinta 1983). At present, the transport proteins known to be involved in carbohydrate phloem loading are mainly sucrose transporters (SUTs) (Riesmeier et al. 1994; Gottwald et al. 2000; Lalonde et al. 2004; Ayre 2011) and SWEETs, a novel class of sugar transporters (Chen et al. 2012). In the apoplastic phloem loading species *Arabidopsis thaliana* and *Solanaceous* species, SWEETs and SUTs participate in the process together, with SWEET transporters moving sucrose across phloem parenchyma cells to apoplast where sucrose is imported into sieve element by SUTs (Braun et al. 2013). In addition, in some plants, phloem loading may simultaneously occur through a variety of different pathways at the same time even within a single vein (Voitsekhovskaja et al. 2009; Slewinski et al. 2013). In certain cases such as biotic or abiotic stress, phloem loading mechanisms in some plants could be modulated (Gil et al. 2011; Lemoine et al. 2013).

RFOs are widespread in plant species, in addition to phloem transport, their physiological functions include carbon storage and protection against various stresses (Sengupta et al. 2015). In seeds, RFOs are prominent soluble sugars and serve as an essential source of easily accessible carbon during  early germination (Downie and Bewley 2001). Stachyose and raffinose are the two important and major soluble sugars among RFOs; stachyose biosynthesis involves the following three steps (Fig. S1): galactinol synthase (GolS, EC 2.4.1.123) catalyses the first step in RFO biosynthesis by synthesizing galactinol from UDP-d-galactose and *myo*-inositol (Saravitz et al. 1987); raffinose is synthesized from sucrose and galactinol by raffinose synthase (RS, EC 2.4.1.82) and *myo*-inositol is produced as the leaving group; stachyose synthesis from raffinose and galactinol is catalyzed by stachyose synthase (STS, EC 2.4.1.67).

Stachyose synthase was originally described in seeds of bean (Tanner and Kandler 1968). Holthaus and Schmitz (1991a) purified STS from the leaves of melon; they also determined the optimal reaction temperature (32 °C), pH (6.8) and K_m_ (3.3 mM for raffinose and 7.7 mM for galactinol) of STS. STS cDNA sequences were initially cloned from seeds of Adzuki Bean. Moreover, the expression of STS rapidly accumulates to high levels during seed development,and its activity is also detected upon seed germination (Peterbauer et al. 1999). The purification, characterization and heterologous expression of STS were reported from developing pea (*Pisum sativum* L.) seeds (Peterbauer et al. 2002).

Although cDNA sequences of STS have been cloned and characterized from several plants (Peterbauer et al. 1999, 2002; Voitsekhovskaja et al. 2009), the gene encoding STS in cucumber (*Cucumis sativus* L.), the classical stachyose-translocating plant (Pharr et al. 1985; Hu et al. 2009), has not been well studied. In this paper, we isolated a stachyose synthase gene (*CsSTS*) from cucumber. Using a combination of histochemical localization, reverse genetics, carbohydrate analysis and abiotic stress treatments, the function of *CsSTS* in symplastic phloem loading, photosynthate distribution and response to low temperature stress were characterized.

## Materials and methods

### Plant materials and growth conditions

Wild-type cucumber plants (*C. sativus* L. cv. Xintaimici) and transgenic cucumber lines (*CsSTS*-RNAi-1, 2, 3 lines and *CsSTS*-OE-1, 2, 3 lines) were used in this study. The plants were grown in greenhouse under normal management. For the carbohydrate analysis of transgenic plants, sampling time was at the end of night (around 4:15 am) and at the end of day (around 8:15 pm) respectively. For low temperature treatment, cucumber seedlings were incubated in a phytotron at 25/18 °C, in a 16 h day/8 h night cycle. At the stage of four to five mature leaves, seedlings were exposed to 6 °C (day/night) and sampled at 0, 12, 24 and 72 h, respectively. The samples were quickly placed in liquid nitrogen and then stored at −80 °C for relative analysis.

### RNA extraction and quantitative RT-PCR analysis

Total RNA samples were extracted using Column Plant RNA out kit (Tiandz, Beijing, China; http://www.tiandz.com), and cDNA was synthesized using PowerScript™ Reverse Transcriptase (Invitrogen, Carlsbad, CA, USA). The cDNA was then used as a template for quantitative real-time PCR (qRT-PCR) analysis, which was performed using SYBR^®^ *Premix Ex Taq*™ from TaKaRa (China) on an Applied Biosystems 7500 RT-PCR system. The cucumber *α-TUBULIN* (*TUA*) served as an internal control, and three biological replicates were performed.

### *CsSTS* gene cloning and phylogenetic analysis

Based on the cucumber genomic sequence, a PCR fragment containing the complete *CsSTS* coding sequence was amplified from cDNA of mature leaves using specific primers (Table S1). The thermal cycling parameters were 95 °C for 5 min, 35 cycles of 95 °C for 30 s, 55 °C for 30 s, and 72 °C for 3 min, and then 72 °C for 10 min. PCR products were cloned into pGEM-T Easy vectors (Promega, Madison, WI, USA) for gene sequencing.

The complete deduced amino acid sequences of related STS proteins in various species were obtained from the National Center for Biotechnology Information (the NCBI http://www.ncbi.nlm.nih.gov) (Fig. S2c). The phylogenetic tree was analyzed by using the neighbor-joining (NJ) method (Saitou and Nei 1987) with MEGA5 software (Tamura et al. 2011).

### Histochemical localization of GUS expression

A pair of PCR primers (Table S1) was designed to amplify 1621 bp of the *CsSTS* promoter. The PCR product was digested with *Hind*III and *Bam*HI and was inserted 5′ of GUS in vector pBI121, yielding construct *pCsSTS*::GUS (Fig. S3a). *Agrobacterium*-mediated transformation was used to transfer the GUS reporter into cucumber. Histochemical localization of the GUS reporter in leaves was performed by incubating the tissues in GUS-staining solution at 37 °C overnight. After staining, samples were cleaned with 75% ethanol and were observed under an anatomical lens. Some samples were prepared as paraffin sections for better observation.

### Immunolocalization

For the immunohistochemical analyses, leaves from cucumber were fixed as described by Wang et al. (2014). After washing with phosphate buffered saline (PBS), the sections were blocked and initially incubated in a 1:200 dilution of rabbit anti-*CsSTS* antibody (CODE458; Beijing B&M Biotech Co., Ltd), followed by an incubation with 1:200 dilution of secondary antibody (anti-rabbit IgG-alkaline phosphatase conjugate; Sigma). After several rinses with PBS, the sections were developed with substrate solution. After fixation, the slides were examined and photographed using microscopy.

### Subcellular localization in cucumber protoplasts and onion epidermal cells

To study the subcellular localization of CsSTS protein, the open reading frame of *CsSTS* cDNA was amplified with gene-specific primers (Table S1) and inserted between the *Sma*I and *Bam*HI sites of the pEZS-NL vector, and empty pEZS-NL vector was used as the control (Fig. S3b). The CsSTS::GFP fusion protein was transiently expressed in onion (*Alliumcepa*) epidermal cells, as described Hayes et al. (2007); the constructs were then introduced into cucumber protoplasts using Huang’s method (2013). GFP fluorescence was visualized with a confocal laser-scanning microscope (Nikon C1, Japan).

### Vector construction and agrobacterium-mediated transformation

To obtain the *CsSTS* over-expression lines, full-length cDNA of *CsSTS* was cloned and inserted the expression vector pBI121 between the *Sma*I and *Bam*HI sites. To build the RNAi construct, two specific fragments of *CsSTS* were amplified using primers with incorporated *Asc*I/*Swa*I sites and *Spe*I/*Bam*HI sites, respectively. These fragments were then inserted into the pFGC1008 vector. Both over-expression and RNAi constructs were transformed into *Agrobacterium tumefaciens* strain LBA4404 respectively. The resulting vectors were transformed into cucumber using the fresh expanding cotyledon disk transformation method as previously described (Sui et al. 2012).

### Stachyose synthase (STS) activity assays

STS was extracted and STS activities were performed according to Peterbauer and Richter (1998) with some modifications. Approximately 0.5 g fresh weight (FW) of leaf samples were ground at 4 °C in 2 mL of chilled extraction buffer (50 mM HEPES-NaOH, pH 7.0; 2 mM DTT; 1% PVP). After 15 min’ standing, the homogenate was centrifuged at 18,000×*g* for 30 min. The supernatant was subject to dialysis at 4 °C for 16 h with buffer containing 25 mM HEPES-NaOH (pH 7.0) and 1 mM DTT, and then used to determine STS activity. The volume of 200 µL reaction mixtures contained 100 mM Hepes-NaOH (pH 7.0), 20 mM β-mercaptoethanol, 40 mM raffinose, 20 mM galactinol, 5 mM MgCl_2_, 4 mM DTT and 50 µL enzyme solution. The mixtures were bathed in water at 30 °C for 3 h and terminated by boiling for 5 min. After cooling, the formation of stachyose was determined by high performance liquid chromatography (HPLC, Agilent 2100 system; Palo Alto, CA, USA) and STS activities were expressed by the content of stachyose. Protein concentration was determined by the method of Bradford with bovine serum albumin as the standard.

### Carbohydrate extraction and analysis

Leaf samples (0.5 g FW) were extensively ground and extracted three times in 5 mL 80% (v/v) ethanol for 30 min at 80 °C. The extracts were combined and decolorized with activated carbon, and then dried at 40 °C in vacuum. The residues were re-dissolved in 1 mL distilled water and then passed through a 0.45 µm filter. Carbohydrate content was determined by high performance liquid chromatography (HPLC, Agilent 2100 system; Palo Alto, CA, USA) using a Sugar-Park column (Waters, 6.5 mm × 300 mm). Samples were eluted with water at 75 °C and a flow rate of 0.5 mL min^−1^; carbohydrates were detected with a refractive index detector. Eluted sugars were identified and quantified from the retention times and peak heights of sugar standards.

### Starch measurement and staining

The fresh sample tissues of 100 mg were taken from the same timeframe. Three biological replicates were done for each time point. A starch measurement kit (Megazyme total starch assay kit) was used to quantify starch content. According to the manufacturer’s protocols of procedure,the starch content was determined by amyloglucosidase/α-amylase method. The leaves were collected after 18 h of dark treatment and decolorized in 80% ethanol at 70 °C. The tissues were immersed in 10% I-KI staining solution for 30 min. After staining, they were washed twice with water for 5 min to remove excess stain. Stained leaves were imaged and photographed under an anatomical lens.

### Measurement of antioxidant enzyme activities

The activity of superoxide dismutase (SOD) was determined by the method as described by Giannopolitis and Ries (1977), according to its ability to inhibit the photoreduction of nitroblue tetrazolium (NBT). The amount of enzyme required to inhibit 50% of the NBT photoreduction was defined as 1 unit of enzyme activity. Catalase (CAT) activity and peroxidase (POD) activity were assayed according the method of Cakmak and Marschner (1992). For the CAT activities, the reaction mixture of 2 mL contained 1700 μL of 25 mM phosphate buffer (pH 7.0), 200 μL of 10 mM H_2_O_2_ and enzyme extract. The decomposition of H_2_O_2_ was followed at 240 nm (coefficient of absorbance, 39.4 mM cm^−1^). For the measurement of POD activities, the reaction mixture contained 100 μL of supernatant, 25 mM phosphate buffer (pH 7.0), 1% guaiacol, 20 mM H_2_O_2_, then reacted under 25 °C, the kinetic changes were determined at 470 nm by ultraviolet-uisible spectrophotometer (coefficient of absorbance, 26.6 mM cm^−1^).

### Determination of lipid peroxidation

The level of lipid peroxidation in the leaves was estimated by measuring MDA content, following the method described by Wu et al. (2012). 0.5 g (FW) leaf material was homogenized in 10 mL of 10% (m/v) trichloroacetic acid (TCA), and then the homogenate was centrifuged at 4000 r min^−1^ for 10 min. The supernatant obtained for 2 mL was added to 2 mL of 0.6% (m/v) thiobarbituric acid (TBA). The mixture was reacted in boiling water for 15 min, and then was centrifuged again after cooling. The absorbance values of homogenate were measured at 450, 532 and 600 nm, respectively.

### The analysis of proline (Pro)

According to Shan’s way (2007), fresh leaf material came from different types was weighed about 0.5 g, then extracted after adding 5 mL of 3% sulfosalicylic acid to them at 100 °C for 10 min, the extracts analyzed for proline content using the acid ninhydrin method. The extract for 2 mL was mixed with 2 mL of acid ninhydrin reagent and 2 mL of glacial acetic acid and heated at 100 °C for 40 min. After cooling, the mixture was reacted with 4 mL toluene and then the absorbance of the organic phase was determined at 520 nm.

### Determination of relative electrical conductivity (REC)

Follow the method described by Liu et al. (2013). The leaves were marinate at room temperature for 12 h and then the electrical conductivity (EC) of the exosmosed solution was measured (C1). The leaves were then boiled for 30 min and the EC was measured again (C2). REC was calculated as (C1/C2) × 100%.

## Results

### Isolation and sequence analysis of *CsSTS*

The isolated full length cDNA of *CsSTS* (GenBank accession No. EF382356) is 2861 bp, and contains a 2595 bp open reading frame encoding a predicted polypeptide of 864 amino acids. Sequence analysis of *CsSTS* cDNA revealed a 109 bp 5′ untranslated region (UTR), a 157 bp 3′ UTR, four exons and three introns (Fig. S2a). The sequence homology of *CsSTS* is 97.6% with *STS* of *Cucumis melo*; it has 71.3, 65.6, and 59.5% sequence homology with those of *Alonsoa meridionalis, P. sativum* and *A. thaliana*, respectively (Fig. S2b). According to the Henrissat glycosyl hydrolase classification system (Henrissat and Davies 2000), all of these proteins belong to glycosyl hydrolase family 36 of the glycosidase super-family GH-D. This super-family is formed by α-galactosidases from families 27 and 36 (Dagnall et al. 1995).

To further understand the evolutionary relationship between *CsSTS* and other *STS* homologues, phylogenetic analysis was performed. It revealed that the *CsSTS* (marked by a black triangle symbol) is highly homologous to STS from melon (*CmSTS*), but is clearly separated from *zea mays* stachyose synthase (*ZmSTS*) and *medicago truncatula* stachyose synthase (*MtSTS*) (Fig. S2c).

### The spatiotemporal expression of *CsSTS*

**Fig. 1:**
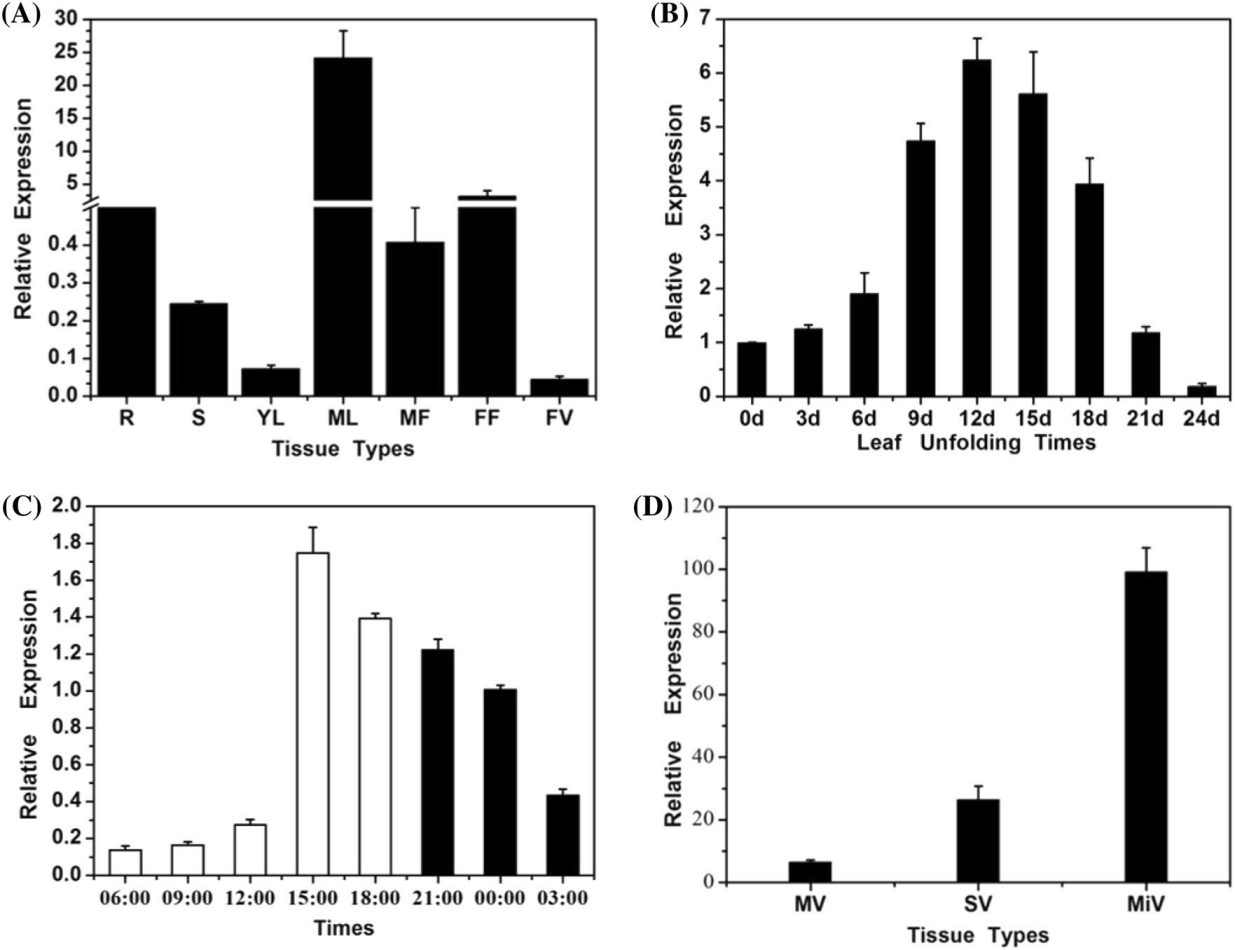
Spatiotemporal expression of *CsSTS*. **a** *CsSTS* expression in different cucumber tissues. **b** *CsSTS* expression during leaf development. **c** Circadian variation of *CsSTS* expression in mature leaves. **d** The expression of *CsSTS* in leaf veins. *R* Root, *S* Stem, *YL* Young leaf, *ML* Mature leaf, *MF* Male flower, *FF* Female flower, *FV* Fruit of the 9th DAF (day after flowering), *d* days, *MV* main vein, *SV* secondary vein, *MiV* minor vein. The data were derived from three biological replicates, and *TUA* in cucumber was used as an internal control. *Error bars* represent standard deviations

Spatiotemporal expression analysis indicated that *CsSTS* expression occurred in all examined tissues; however, the transcript levels were the highest in mature leaves and were very low in other organs such as roots, stems, young leaves, male and female flowers and fruits (Fig. 1a). After leaf unfolding, relative expression levels of *CsSTS* increased gradually to a maximum on day 12, and then declined rapidly (Fig. 1b). Over 24 h, *CsSTS* expression levels were lower in the morning and higher in the afternoon, peaking around 15:00 in the afternoon and then declining (Fig. 1c). In addition, *CsSTS* was expressed mainly in minor vein phloem of leaves (Fig. 1d), revealing that *CsSTS* was not only responsible for stachyose synthesis, but may be also related to phloem loading in source leaves.

### The histochemical and subcellular localization of *CsSTS*

**Fig. 2:**
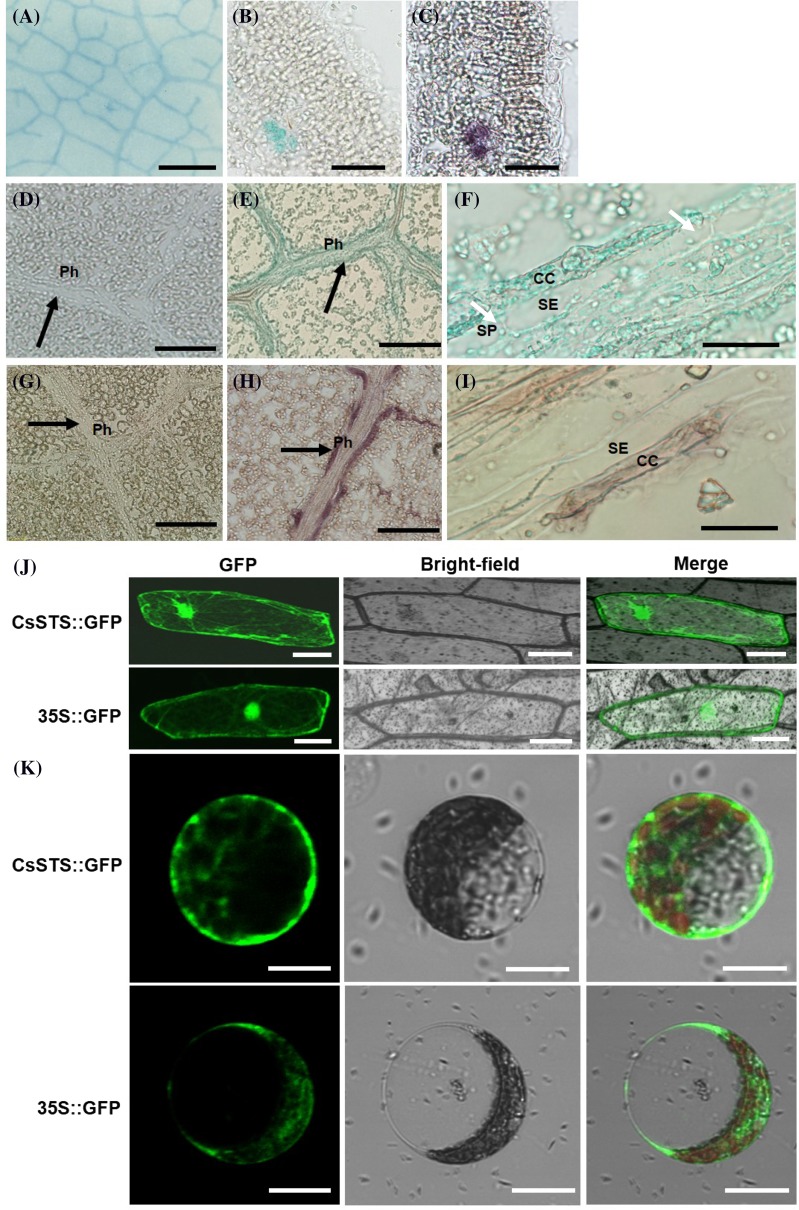
Histochemical and subcellular localization of *CsSTS*. Histochemical analysis of *pCsSTS*::GUS plants (**a**,** b**,** d**,** e**, **f**); immunolocalization of *CsSTS* in cucumber leaves (**c**,** g**,** h**, **i**). The section of the leaf without GUS expression (**d**); The control sections treated with pre-immune serum (**g**). Longitudinal sections (**d**–**i**); transverse sections (**b**, **c**). *Black arrows* indicate the Ph in (**d**,** e**,** g**,**h**); *white arrows* in **f** indicate the SP. Subcellular localization of *CsSTS* protein in onion epidermal cells (**j**); Subcellular localization of *CsSTS*::GFP fusion protein in cucumber mesophyll protoplasts (**k**). *CC* companion cells, *SP* sieve plate, *SE* sieve element, *Ph* phloem. *Scale bars* denote: **a** 2 mm, **b** and **c** 100 μm; **d**, **e**, **g**, **h** 200 μm; **f**, **i**, **j** 50 μm; **k** 20 μm

Based on high *CsSTS* expression levels in leaves, we examined the detailed expression pattern of *CsSTS* in cucumber leaves by histochemical analysis of *pCsSTS*::GUS plants (Fig. 2a, b, d, e, f) and immunolocalization (Fig. 2c, g, h, i). The results indicated that *CsSTS* was expressed mainly in minor veins (Fig. 2a–c). Further analysis demonstrated *CsSTS* expression in the bicollateral phloem of the vascular bundle in the minor veins of leaves, but not in vessel elements (Fig. 2e, h). The close-up of Fig. 2e, h clearly shows *CsSTS* targeting to companion cells (CC) (Fig. 2f, i). The subcellular localization of *CsSTS* was determined by expressing the *CsSTS*::GFP fusion protein in chloroplast-free epidermal cells of onion (*Allium cepa*) (Fig. 2j) and cucumber protoplasts (Fig. 2k), with empty GFP containing pEZS-NL serving as the control. The results showed that *CsSTS*::GFP was targeted to the plasma membrane, nucleus and cytosol of epidermal cells of onion, and the plasma membrane and cytosol of cucumber protoplasts.

### Construction of *CsSTS* -OE and *CsSTS* -RNAi lines

**Fig. 3:**
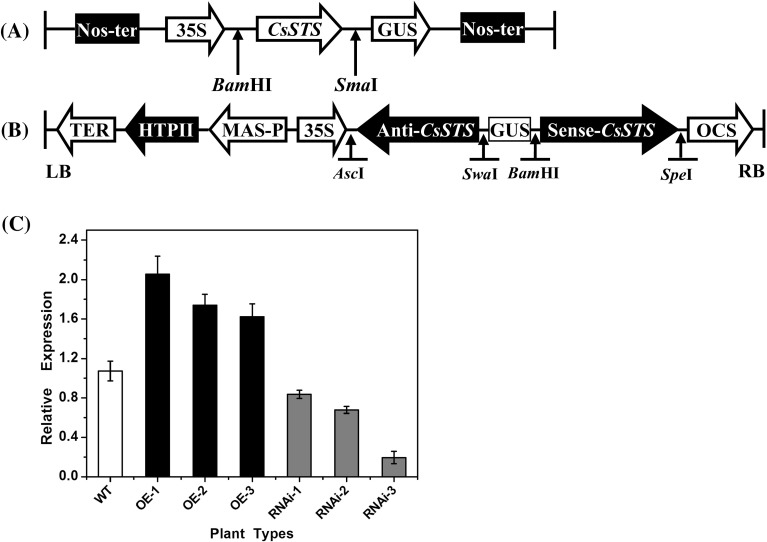
Construction of *CsSTS*-OE and *CsSTS*-RNAi lines. **a** Schematic diagram of *STS* gene over expression vector. **b** Diagram of the *CsSTS*-RNAi construct. **c** The relative expression analyses of *CsSTS* in WT, *CsSTS*-OE and *CsSTS*-RNAi lines by qRT-PCR. *TUA* in cucumber was used as an internal control, and the experiments were repeated in three intervals. *WT* wild-type, *OE* over expression lines; *RNAi* RNAi lines. *Error bars* indicate standard deviations

In order to further confirm the biological function of *CsSTS*, the entire *CsSTS* coding sequence was amplified by PCR and inserted into the pBI121 plant expression vector in the sense orientation (Fig. 3a). At the same time, a double-strand RNAi vector was constructed containing the *CsSTS* specific sequence of under control of the 35 S promoter (Fig. 3b). Then, the constructs were introduced by *Agrobacterium*-mediated transformation into cucumber cotyledons for expression, and 20 transgenic plants were obtained. The expression level of *CsSTS* increased in OE lines and decreased in RNAi lines, suggesting that the *CsSTS* sequences were integrated into cucumber genome and functional (Fig. 3c). Based on *CsSTS* expression levels by qRT-PCR, three *CsSTS*-RNAi (RNAi-1, -2, -3) lines and three *CsSTS*-OE (OE-1, -2, -3) lines were selected for further study (Fig. 3c).

### Carbohydrate analysis of transgenic plants

**Fig. 4:**
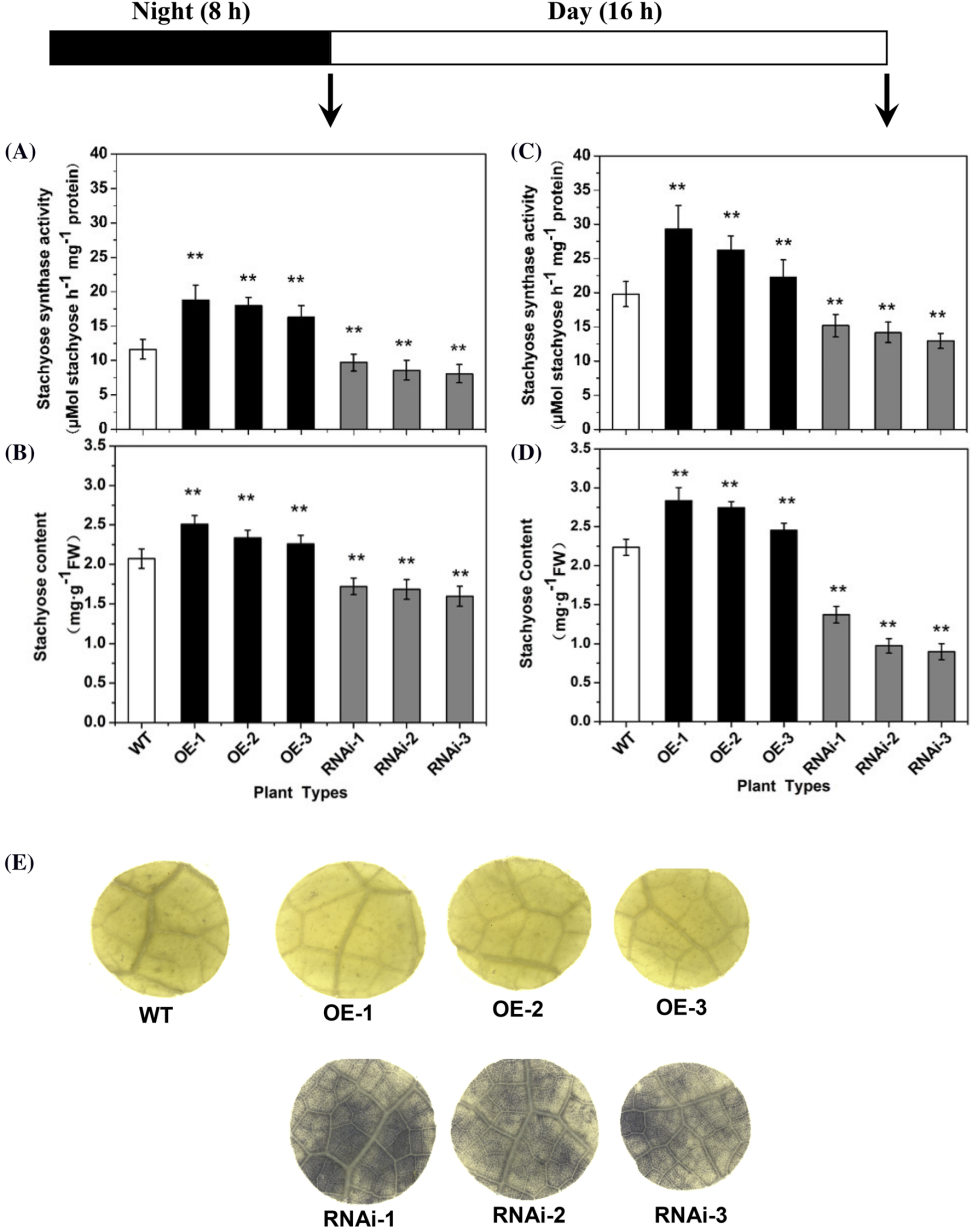
The STS activities, stachyose contents, starch staining of leaves in transgenic and wild-type cucumber. The STS activities (**a**, **c**) and stachyose contents (**b**, **d**) at the end of night (**a**, **b**) and at the end of day (**c**, **d**) respectively. The sampling time was around 4:15 am (**a**, **b**) and 8:15 pm (**c**, **d**), respectively. Staining of starch was done in wild type and transgenic plants after 18 h of dark treatment (**e**). *WT* wild-type, *OE* over expression lines, *RNAi* RNAi lines. Each value is the mean ± standard deviation of three replicates. Significance differences were determined by Duncan’s test (*P < 0.05, **P < 0.01)

Compared with wild-type plants, both *CsSTS*-OE and *CsSTS*-RNAi transgenic lines seemed to have normal phenotypes; however, STS activities and stachyose contents in leaves of both transgenic lines and wild-type plants had marked differences (Fig. 4). Analysis of leaf STS activities showed that when compared to wild type, the STS activities were lower in *CsSTS*-RNAi lines and were higher in *CsSTS*-OE lines, especially at the end of night (Fig. 4a, c). The stachyose contents were lower in *CsSTS*-RNAi lines and higher in *CsSTS*-OE lines relative to wild-type, especially at the end of day (Fig. 4b, d).

Due to inhibition of stachyose synthesis and export in *CsSTS*-RNAi lines, there theoretically should be some starch accumulation. To this end, leaves detached from wild type and transgenic plants were placed in the dark for 18 h, and then stained by iodine solution. The results revealed only trace amounts of accumulated starch in the *CsSTS*-OE and wild type lines, but there was still a small amount of starch in the leaves of the *CsSTS*-RNAi lines, suggesting that starch reserves in the *CsSTS*-RNAi lines remained even after 18 h of darkness (Fig. 4e). The above results demonstrated the promotion of stachyose synthesis and transport in *CsSTS*-OE lines, preventing starch accumulation. Conversely, stachyose synthesis and export were inhibited in *CsSTS*-RNAi lines, which accumulated a small amount of starch. These revealed that *CsSTS* plays a key role in phloem loading and transport of carbohydrate in cucumber leaves.

**Fig. 5:**
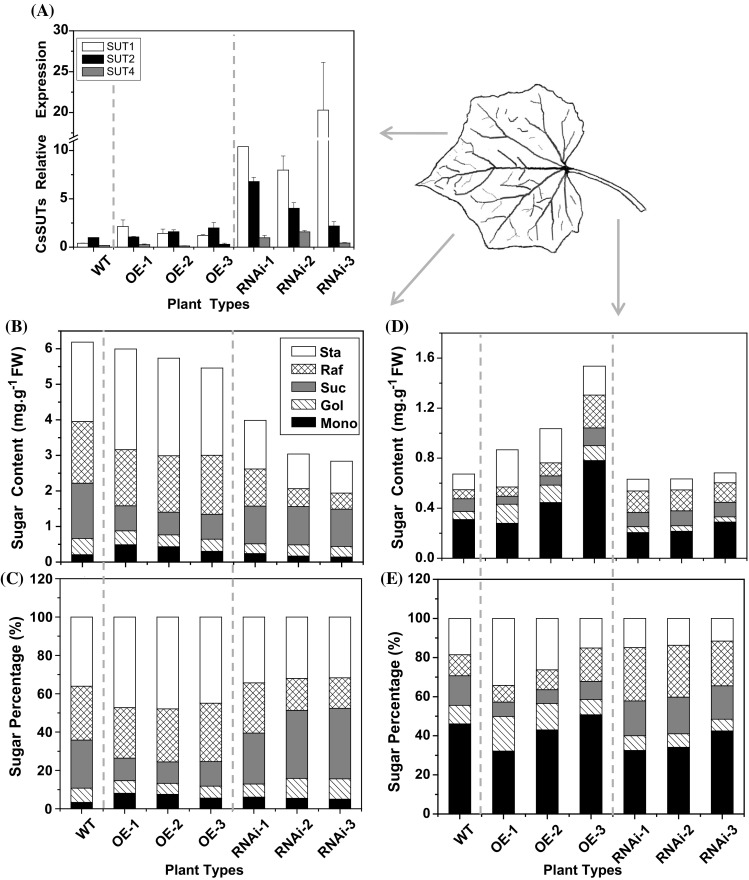
The expression of sucrose transporters in leaves (**a**) and sugar contents and percentages in leaves (**b**, **c**) and petioles (**d**, **e**) of transgenic and wild-type cucumber plants. *WT* wild-type, *OE* over expression plants, *RNAi* RNA interference lines, *Sta* stachyose, *Raf* raffinose, *Suc* sucrose, *Gol* galactinol, *Mono* monosaccharide. *TUA* was used as an internal control and the experiments were repeated in three intervals. *Error bars* indicate the standard deviations

The sucrose transporters (SUTs) play an essential role in apoplastic phloem loading of sucrose (Riesmeier et al. 1994; Gottwald et al. 2000; Lalonde et al. 2004). There were three sucrose transmembrane transporters in cucumber named *CsSUT1, CsSUT2* and *CsSUT4*, respectively. It is unknown whether cucumber SUT participates in phloem loading of cucumber which is generally classified as symplastic loaders. Interestingly, we found obvious differences in expression levels of the three cucumber sucrose transporters (*CsSUTs*) in transgenic lines and wild type plants. Compared with wild type, the expression level of *SUT* 1, 2 and 4 were significantly higher in *CsSTS*-RNAi lines; but in *CsSTS*-OE lines, their expression levels were similar to those of wild type lines (Fig. 5a). Accordingly, sucrose levels in the leaves of *CsSTS*-RNAi lines were reduced to a certain extent (Fig. 5b), and sucrose content (Fig. 5d) and distribution percentage (Fig. 5e) in the petiole increased slightly. Presumably, after suppression of stachyose synthesis, loading and export in *CsSTS*-RNAi lines, expression of *SUTs* were up-regulated to compensate via apoplastic phloem loading through SUT transmembrane transportation. In addition, compared with wild-type plants, raffinose content and distribution percentage obviously decreased in leaf blades (Fig. 5b, c), but significantly increased in petioles (Fig. 5d, e). It’s likely that more amount of raffinose was loaded and transported to the petiole in *CsSTS*-RNAi plants.

### *CsSTS* involved in response to low temperature stress

**Fig. 6:**
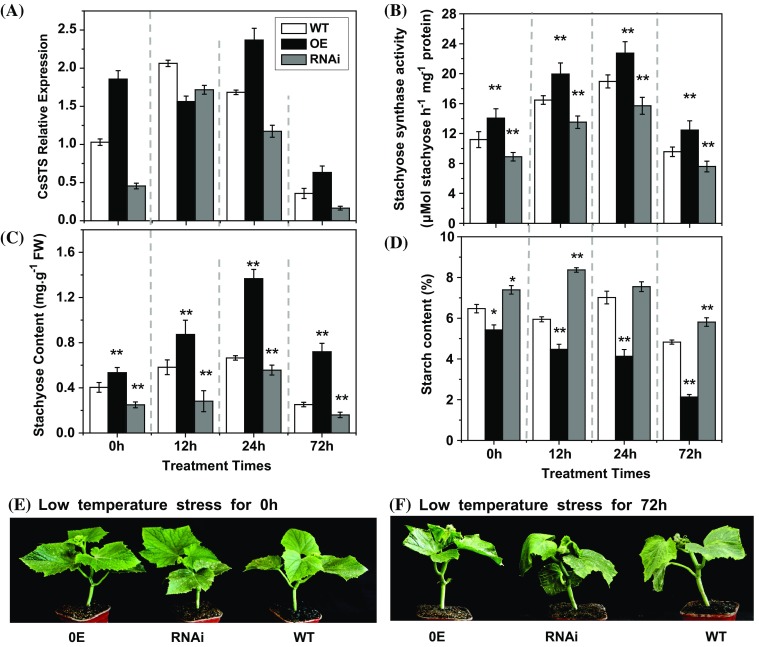
The effect of low temperature (6 °C) on gene expressions, activities of STS, stachyose contents, starch contents and phenotypes of leaf in transgenic and wild-type cucumber seedlings. **a** The *CsSTS* expression in leaves. **b** The activities of STS. **c** Stachyose contents. **d** Starch contents. The phenotypes of different plant types at 0 h (**e**) and 72 h (**f**) after low temperature treatment. *WT* wild-type, *OE* over expression lines, *RNAi* RNA interference lines, *h* hours. *TUA* was used as an internal control. Each value is the mean ± standard deviation of three replicates. Significance differences were determined by Duncan’s test (*P < 0.05, **P < 0.01)

**Fig. 7:**
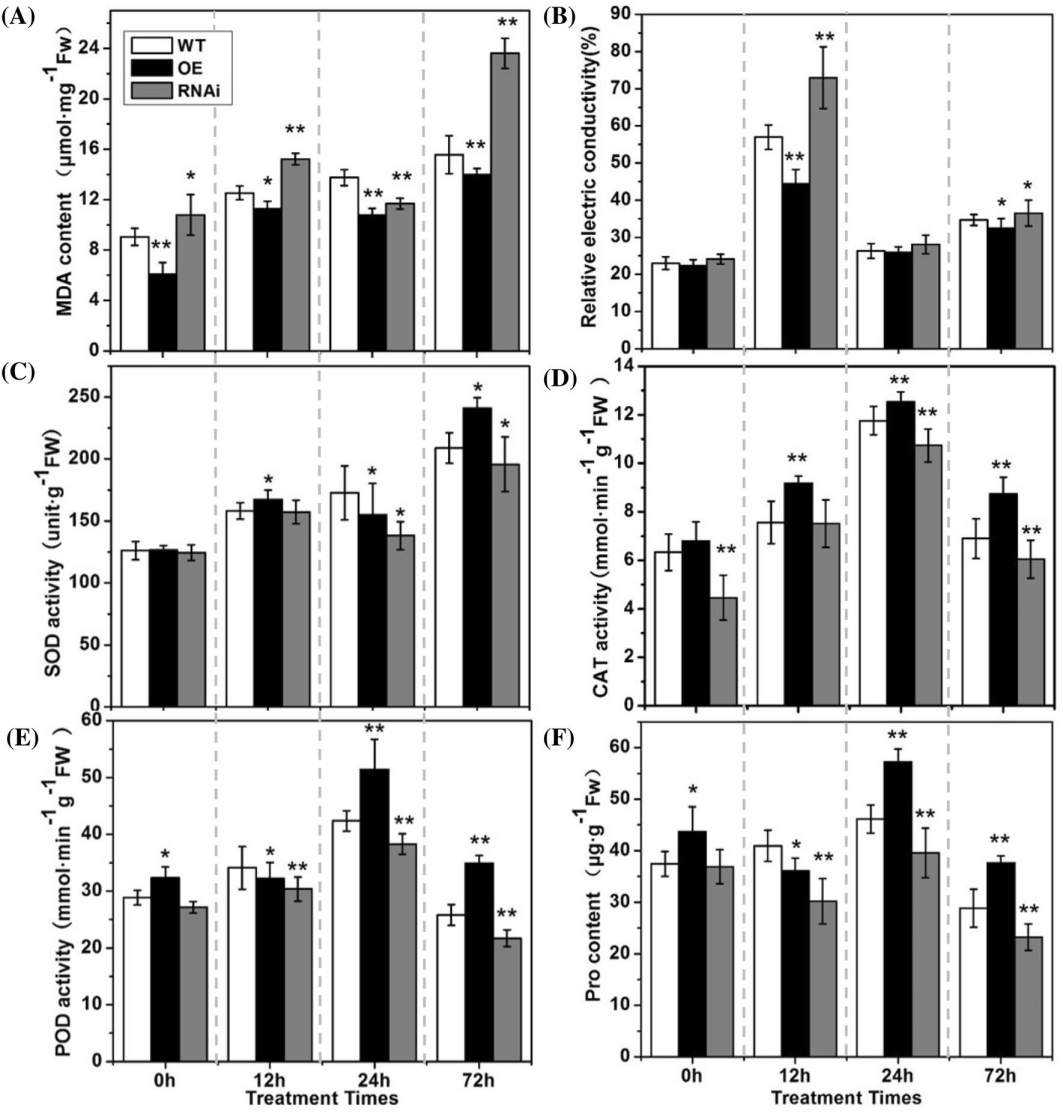
The effects of low temperature (6 °C) on membrane peroxidation, activities of antioxidant enzymes and osmotic substance between the different plant types of cucumber seedlings. **a** Malondialdehyde (MDA) contents. **b** Relative electric conductivities. **c** Superoxide dismutase (SOD) activities. **d** Catalase (CAT) activities. **e** Peroxidase (POD) activities. **f** Proline contents. *WT* wild-type, *OE* over expression lines, *RNAi* RNA interference lines, *h* hours. Each value is the mean ± standard deviation of three replicates. Significance differences were assessed by Duncan’s test (*P < 0.05, ** *P* < 0.01)

Cucumber is a thermophilic crop, and easily suffers damage from low temperature stress. It is unknown whether cucumber stachyose synthase is involved in the response to low temperature stress. To this end, the seedlings mutant at the stage of four to five mature leaves were selected and treated with 6 °C (day/night) low temperature stress in order to further study the function of *CsSTS* (Figs. 6, 7). When suffered low temperature stress, *CsSTS* expression was up-regulated (Fig. 6a) and enzyme activities (Fig. 6b) and stachyose contents (Fig. 6c) increased gradually in wild type plants and transgenic plants after 24 h, and declined after 72 h treatment. The leaves of *CsSTS*-RNAi lines experienced obvious wilting and sagging, whereas wild-type plants also had slight wilting symptoms, but the *CsSTS*-OE lines seemed to be normal in phenotype after 72 h low temperature treatment (Fig. 6e, f). During cold treatment, *CsSTS* expression, STS activities, and stachyose contents were always higher in *CsSTS*-OE lines and lower in *CsSTS*-RNAi lines relative to wild type plants (Fig. 6a–c). Stachyose content was significantly higher in *CsSTS*-OE lines than that in *CsSTS*-RNAi lines and wild type plants (Fig. 6c), suggesting that higher stachyose content contributed to enhanced low temperature stress tolerance. Moreover, a large amount of starch was accumulated in the leaves of *CsSTS*-RNAi lines during the stress treatment (Fig. 6d), which may reflect changes in RFO metabolic balance. By comparison, the *CsSTS*-OE lines and wild type had higher stachyose levels (Fig. 6c) and reduced accumulation of starch (Fig. 6d). These results suggest that stachyose plays an important role in cold tolerance of cucumber plants.

Physiologically, malondialdehyde (MDA) contents (Fig. 7a) and the relative electric conductivities (Fig. 7b) in the wild type and the mutant seedlings showed increase trend, especially in the *CsSTS*-RNAi lines during low temperature stress, which revealed that the cell membrane was probably damaged by reactive oxygen species (ROS) under low temperature stress. Accordingly, the activities of the reactive-oxygen-scavenging enzyme, such as superoxide dismutase (SOD) (Fig. 7c), catalase (CAT) (Fig. 7d) and peroxidase (POD) (Fig. 7e) in cucumber seedlings, increased gradually during low temperature stress, especially in *CsSTS*-OE lines. Moreover, the proline as an important osmotic regulation substance, its contents in the leaves of *CsSTS*-RNAi plants was lower than in the leaves of *CsSTS*-OE plants (Fig. 7f). These results showed that the reactive-oxygen-scavenging enzyme systems and the proline in cucumber seedlings play an important role to reduce low temperature stress damage.

## Discussion

Raffinose family oligosaccharides (RFOs) metabolism is a complex process in plants which involves many enzymes. Among them, STS is one of the most important enzymes in the RFO biosynthetic pathway (Fig. S1), and stachyose and raffinose are the two main members of RFOs. Cucumber (*C. sativus* L.) is a stachyose-translocating plant (Pharr et al. 1985; Hu et al. 2009). Compared with the most sucrose-transporting plants, the source to sink carbohydrate metabolism of stachyose-transporting plants is more complex. In early report, expression of *STS* in *Cucurbitaceae* was detected mainly in leaves and seeds (Hendrix 1968). In *Scrophulariaceae*, expression levels of *STS* were the highest in mature leaves and cotyledons, followed by young leaves; no hybridization signal was detected for RNA from stems, roots, flowers and fruits (Voitsekhovskaja et al. 2009). Similarly, in cucumber plants, the relative expression of *STS* was highest in mature leaves, especially in the minor veins (Fig. 1a, d).

A more comprehensive localization of stachyose synthase was carried out by Holthaus and Schmitz (1991b). According to the indirect evidence of the distribution of stachyose, raffinose, galactinol and enzyme activities of STS, it was thought that stachyose was synthesized mainly in minor veins of mature leaves and in seeds. Furthormore, stachyose synthase had been detected in intermediary cells (IC) of minor vein phloem of *Cucumis melo* (Holthaus and Schmitz 1991b) and *A. meridionalis* (Voitsekhovskaja et al. 2009). Generally, there are three types of companion cells (CC) in stachyose-transporting species: intermediary cells (IC), transfer cells (TC) and ordinary companion cells (OCC). The main difference between IC cells and the other two types of companion cells is the presence of many plasmodesmata (Turgeon et al. 1975; Volk et al. 1996). According to the polymer trap mechanism (Turgeon 1996), a number of symplastic phloem loading species synthesize RFOs in the ICs of minor-vein phloem (Holthaus and Schmitz 1991b; Haritatos et al. 1996; Büchi et al. 1998; Voitsekhovskaja et al. 2009). In our histochemical analysis of *pCsSTS*::GUS and immunolocalization of *CsSTS* in cucumber leaves (Fig. 2), *CsSTS* was targeted to the companion cells (CC) of the minor-vein phloem. Data in this paper support the model that *STS* is targeted to IC, and is involved in symplastic phloem loading. The data provide further structural and anatomical details in cucumber, a typical *Cucurbitaceae* plant, refining the polymer trap model theory. Furthermore, based on localization results of *CsSTS*::GFP fusion protein in cucumber mesophyll protoplasts and onion epidermal cells, it is revealed that *CsSTS* is targeted to plasma membrane and cytosol (Fig. 2). In comparing with previous results, the localization results from our experiment were clearer in anatomy, more intuitive and accurate in ultrastructure and subcellular level.

Sucrose and starch are the primary products of photosynthesis in the leaves of most plants (Kötting et al. 2010). When phloem loading is inhibited in the symplastic or apoplastic pathways, starch will accumulate in leaves. Similar results were obtained in this paper when expression of *CsSTS* was altered compared with wild type lines, *CsSTS*-RNAi lines accumulated starch in leaves and *CsSTS*-OE has significant reductions in stored starch (Fig. 4e), suggesting that phloem loading in *CsSTS*-RNAi lines was inhibited to some extent. At the same time, STS activities and stachyose contents were higher in the *CsSTS*-OE plants and lower in *CsSTS*-RNAi lines relative to wild type, especially at the end of night. These results revealed that export of photosynthate was suppressed to some extent in *CsSTS*-RNAi lines and starch was accumulated. However, alteration of *CsSTS* expression did not seem to influence plant phenotype.

The consistent association of RFOs with intermediary cells suggests an integral role of RFOs synthesis for the phloem-loading mechanism (Ashlee and Turgeon 2007). The ultrastructural and molecular studies provide the experimental data to classify cucurbits as symplastic loaders (Mitchell and Madore 1992; Haritatos et al. 1996). In this study, the levels and distribution percentage of raffinose in the petioles of *CsSTS*-RNAi lines were significantly elevated; this may compensate partly for a lack of stachyose synthesis in RNA interference transgenic plants (Fig. 5). The results further confirm that the synthesis of RFOs in leaves is necessary for efficient phloem loading and transport, consistent with the polymer trapping model. This also revealed that in cucumber a symplastic phloem loading pathway dominates.

Many studies indicate that sucrose transporters (SUTs) are involved in apoplastic phloem loading (Lalonde et al. 2004; Braun and Slewinski 2009). Accordingly, down-regulating of sucrose transporter *VpSUT*1 in the stachyose-transporting species *Verbascum phoeniceum* did not inhibit phloem loading (Zhang and Turgeon 2009). In this paper, we found up-regulation of *CsSUT* 1, 2 and 4 in *CsSTS*-RNAi lines and their expressions were obviously higher than those of both the wild-type and *CsSTS*-OE lines, while there were no significant differences between *CsSTS*-OE and wild type plants (Fig. 5a). Although polymer trapping is the primary phloem loading strategy in cucurbits, the regulation of sucrose transporter expression levels in leaves and sucrose content in petioles of *CsSTS*-RNAi lines suggests that transgenic cucumber with interference expression of *CsSTS* probably contain the machinery for apoplastic phloem loading when the stachyose synthesis was blocked obviously. This result was similar to that of Gil et al. (2011), where melon plant *CmSUT1* transcript expression in vascular bundles of minor veins increased significantly upon infection with cucumber mosaic virus (CMV), and was associated with elevated sucrose content in phloem sap collected from source-leaf petioles. Furthermore, *AmSTS1*, a stachyose synthase in the stachyose-translocating plant *A. meridionalis*, was expressed in the IC but not in the OCC of the same minor vein, while sucrose transporter *AmSUT1* protein was present in OCC but not in the IC (Voitsekhovskaja et al. 2009). This indicates that both symplastic and apoplastic pathways can function simultaneously during phloem loading and these pathways are separated at the level of different sieve element-companion cell complexes combined in their phloem endings. However, the histochemical localization of cucumber sucrose transporters and their role in the phloem loading were elusive. Moreover, the “mixed” or “heterogeneous” loading mechanism of cucumber will need to be further confirmed with anatomical, physiological and molecular biology studies in the near future.

When plants are subjected to cold stress, soluble carbohydrates are differentially accumulated in vegetative tissues, and these cryoprotectant carbohydrates protect biological tissues from freezing damage (Imanishi et al. 1998; Zuther et al. 2004, 2012; Peters and Keller 2009). The RFO biosynthetic pathway is essentially an extension of the inositol metabolic pathway (Loewus and Murthy 2000; Sengupta et al. 2012). In plants, RFOs do not appear to have a direct functional relationship to stress amelioration under natural condition (Sengupta et al. 2015); the main RFO members, raffinose and stachyose, are stored in high concentrations and are not subject to rapid depletion under low temperature in order to increase cold tolerance of plants (Hinesley et al. 1992; Bachmann et al. 1994; Haab and Keller 2002; Tapernoux-Lüthi and Keller 2004).

The degree of low temperature damage to leaves in diverse plant types differ greatly. Cucumbers originate in the subtropics and belong to thermophilic crops, and they easily suffer damage under low temperature stress. Many experiments offer clarification for the roles of RFOs in stress protection, especially that of raffinose, which is ubiquitous in plants and has been implicated in tolerance to freezing in various species; examples include *Vitis vinifera, Medicago sativa* and even conifers (Castonguay et al. 1995; Strimbeck et al. 2007; Grant et al. 2009). Some studies have shown that RFOs may function to protect the thylakoid but not the plasma membrane during freezing in *Arabidopsis* (Iftime et al. 2011). Of course, there are many physiological and biochemical processes and reactions in the synthesis and transportation of stachyose. In this process, many substrates, intermediate products and enzymes are involved, and these different enzymes are regulated by their own genes. These genes, such as *GolS* (galactinol synthase), *RS* (raffinose synthetase) and *STS*, have their own specific roles and localizations within plant tissue; some exist as members of families genes and have diverged to perform specific but related functions such as the response to biotic and abiotic stresses. In our study, the level of membrane lipid peroxidation and cell membrane permeability, activities of SOD, CAT and POD protective enzymes and the content of osmotic adjusting materials in wild type or transgenic seedling increased gradually as suffering 6 °C low temperature stress (Fig. 7). Moreover, after 72 h low temperature treatment, the leaves of *CsSTS*-RNAi lines were markedly wilted and sagged (Fig. 6e, f). This was likely related to the reduced expression of *CsSTS* along with reduced STS enzyme activity and stachyose content in *CsSTS*-RNAi seedlings relative to wild-type or especially *CsSTS*-OE lines (Fig. 6a–c). To be sure, *CsSTS* suppression significantly increased the peroxidation of membrane-lipid, decreased the stabilities of cell membrane system, and reduced the ability of resistance and adaption of cucumber plants to low temperature stress.

In summary, our study showed that *CsSTS* not only affects stachyose synthesis, phloem loading and the distribution of carbohydrates in the leaves, but also enhances the tolerance to low temperature stress.

## Electronic supplementary material

Below is the link to the electronic supplementary material.

Supplementary material 1 (DOCX 17 KB)

Supplementary material 2 (DOCX 1934 KB)

Supplementary material 3 (DOCX 23 KB)

Supplementary material 4 (DOCX 16 KB)
