# Supplementary material for: Suppression of cucumber stachyose synthase gene (CsSTS) inhibits phloem loading and reduces low temperature stress tolerance
Source: Plant Mol Biol. 2017 Jun 12;95(1):1–15. doi: 10.1007/s11103-017-0621-9 (PMC5594042; doi:10.1007/s11103-017-0621-9)
Supplement: Supplementary file 1 — Supplementary material 1 (DOCX 17 KB) [file 11103_2017_621_MOESM1_ESM.docx]

**Fig. S1** The biosynthetic pathway of Raffinose family oligosaccharides (RFOs) in plant.
