# Supplementary material for: Suppression of cucumber stachyose synthase gene (CsSTS) inhibits phloem loading and reduces low temperature stress tolerance
Source: Plant Mol Biol. 2017 Jun 12;95(1):1–15. doi: 10.1007/s11103-017-0621-9 (PMC5594042; doi:10.1007/s11103-017-0621-9)
Supplement: Supplementary file 2 — Supplementary material 2 (DOCX 1934 KB) [file 11103_2017_621_MOESM2_ESM.docx]

**(A)**


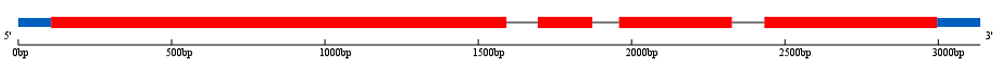


**(B)**


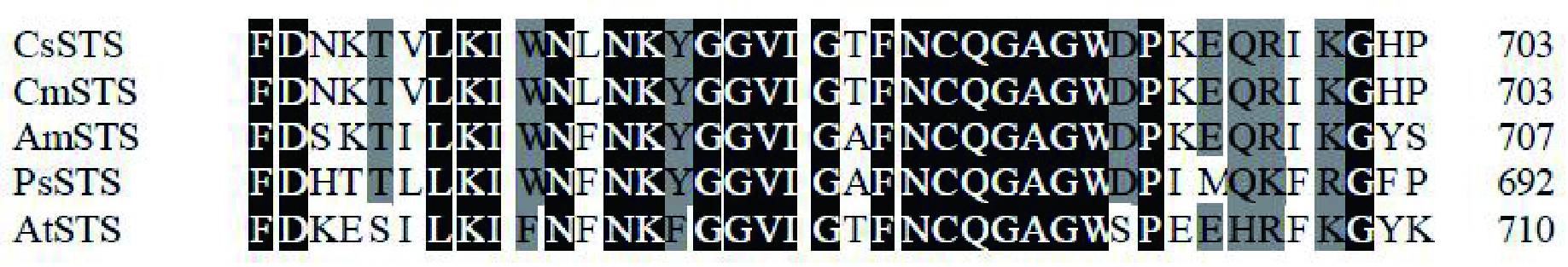


**(C)**


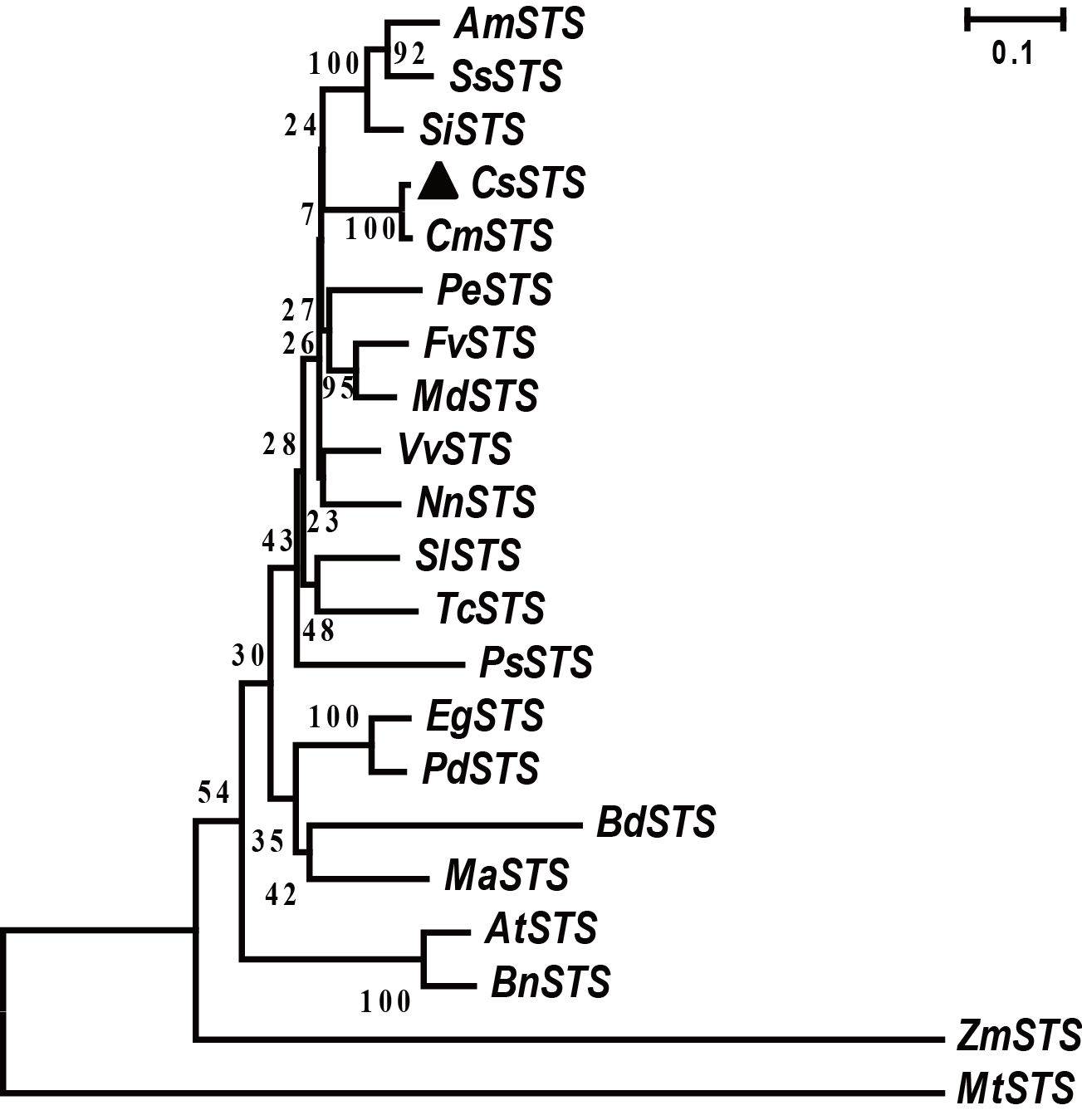


**Fig. S2** The structure (**a**), sequence alignment (**b**) and phylogenetic tree (**c**) analysis of *STS* gene in cucumber. Exons, introns and upstream/ downstreams in **(a)** are represented by red boxes, black lines and blue boxes, respectively. The phylogenetic tree was constructed using the Neighbor–Joining (NJ) method through MEGA5 software. Bars = 0.1. The species , *STS* abbreviations and amino acid sequence accession numbers are: *Alonsoa meridionalis* - AmSTS (CAD31704), *Stachys sieboldii* - SsSTS (CAC86963), *Sesamum indicum* - SiSTS (XP_011079533), *Cucumis sativus* - CsSTS (ABQ53598), *Cucumis melo* - CmSTS (XP_008451468), *Populus euphratica* - PeSTS (XP_011042279), *Fragaria vesca subsp. vesca* - FvSTS (XP_004288541), *Malus x domestica* - MdSTS (XP_008381428), *Vitis vinifera* - VvSTS (XP_002271259), *Nelumbo nucifera* - NnSTS (XP_010259226), *Solanum lycopersicum* - SlSTS (XP_004229378), *Theobroma cacao* - TcSTS (XP_007051146), *Pisum sativum* - PsSTS (CAC38094), *Elaeis guineensis* - EgSTS (XP_010931766), *Phoenix dactylifera* - PdSTS (XP_008797480), *Brachypodium distachyon* - BdSTS (XP_003560876), *Musa acuminata subsp. malaccensis* - MaSTS (XP_009383790), *Arabidopsis thaliana* - AtSTS (NP_192106), *Brassica napus cultivar* - BnSTS (ADQ20113), *Zea mays* - ZmSTS (NP_001152291), *Medicago truncatula* - MtSTS (AES79650).
