# Supplementary figures and images for: Suppression of cucumber stachyose synthase gene (CsSTS) inhibits phloem loading and reduces low temperature stress tolerance
Source: Plant Mol Biol. 2017 Jun 12;95(1):1–15. doi: 10.1007/s11103-017-0621-9 (PMC5594042; doi:10.1007/s11103-017-0621-9)

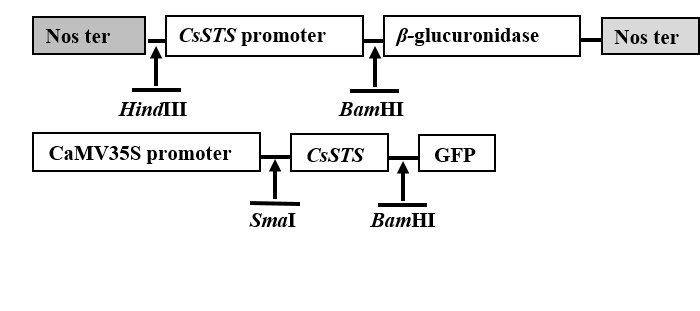


**(B)**

**(A)**

**Fig. S3** Diagrams of the *CsSTS*-promoter::GUS (**a**) and *CsSTS*::GFP (**b**) construct.

Supplement: Supplementary file 3 — Supplementary material 3 (DOCX 23 KB) [file 11103_2017_621_MOESM3_ESM.docx]
