# Supplementary material for: Suppression of cucumber stachyose synthase gene (CsSTS) inhibits phloem loading and reduces low temperature stress tolerance
Source: Plant Mol Biol. 2017 Jun 12;95(1):1–15. doi: 10.1007/s11103-017-0621-9 (PMC5594042; doi:10.1007/s11103-017-0621-9)
Supplement: Supplementary file 4 — Supplementary material 4 (DOCX 16 KB) [file 11103_2017_621_MOESM4_ESM.docx]

**Table S1** Primer sequences and their uses. (F-forward primers; R-reverse primers.)

| **Primers** | **Restriction sites and protect bases** | **Sequence (5'→ 3')** | **Used for** |
| --- | --- | --- | --- |
| *CsSTS*-F | **————** | ATGGCACCTCCAAATGACCC | Cloning of *CsSTS* |
| *CsSTS*-R | **————** | TCAAAAGAAAATATCCAAA TTAGAAACT |  |
| *CsSTS*-OE-F | *BamH* I（ CG**GGATCC**） | ATGGCACCTCCAAATGACCC | *CsSTS* overexpression construct |
| *CsSTS*-OE-R | *Sma* I（ TCC**CCCGGG**） | TCAAAAGAAAATATCCAAA TTAGAAACT |  |
| *CsSTS*-RNAi-1-F | *BamH* I（ CG**GGATCC**） | GATCAAGAGCCATCTGTGGAGGTC | *CsSTS-*RNAi  construct |
| *CsSTS*-RNAi-1-R | *Spe*I（ GG**ACTAGT**） | TCTAGTGGGGAGGGCAAAATGT |  |
| *CsSTS*-RNAi-2-F | *Swa*I（**ATTTAAAT**） | GATCAAGAGCCATCTGTGGAGGTC |  |
| *CsSTS*-RNAi-2-R | *Asc*I（TT**GGCGCGCC**） | TCTAGTGGGGAGGGCAAAATGT |  |
| *CsSTS* -GFP-F | *Sma* I（ TCC**CCCGGG**） | ATGGCACCTCCAAATGACC | *CsSTS*::GFP construct |
| *CsSTS* -GFP-R | *BamH* I（ CG**GGATCC**） | AAAGAAAATATCCAAATTAGAAACTCC |  |
| *CsSTS*-GUS-F | *Hind* III（ CC**AAGCTT** ） | TGAAAAATGTCTCAGGCAC | *CsSTS*-promoter::GUS construct |
| *CsSTS*-GUS-R | *BamH* I（ CG**GGATCC**） | CAATGAAAGACAAACAACCC |  |
| *TUA*-F | **————** | ACGCTGTTGGTGGTGGTAC | Internal controls in  cucumber |
| *TUA*-R | **————** | GAGAGGGGTAAACAGTGAATC |  |
| q-*CsSTS*-F | **————** | CAACTGTCAAGGAGCAGGGTGGG | qRT-PCR of *CsSTS* |
| q-*CsSTS*-R | **————** | AGCGGTTCGGATTTTGGGGT |  |
| q-*CsSUT1*-F | **————** | CGTGGTTACAAAGGTTGCTGAG | qRT-PCR of *CsSUT1* |
| q-*CsSUT1*-R | **————** | GCGGATACGATGAACTGTGGA |  |
| q-*CsSUT2*-F | **————** | TTCTAACTCCCTATATTCAGACGCTC | qRT-PCR of *CsSUT2* |
| q-*CsSUT2*-R | **————** | GCAATGCTCCTTTGTATCTCCTAA |  |
| q-*CsSUT4*-F | **————** | GTCCTCCCTGGTTGTAGAAGAAAG | qRT-PCR of *CsSUT4* |
| q-*CsSUT4*-R | **————** | CTCTACCCATCCAATCAGTATCAAAG |  |
